# The role of sodium channels in sudden unexpected death in pediatrics

**DOI:** 10.1002/mgg3.1309

**Published:** 2020-05-25

**Authors:** Anne M. Rochtus, Richard D. Goldstein, Ingrid A. Holm, Catherine A. Brownstein, Eduardo Pérez‐Palma, Robin Haynes, Dennis Lal, Annapurna H. Poduri

**Affiliations:** ^1^ Department of Neurology Boston Children's Hospital and Harvard Medical School Boston MA USA; ^2^ Robert’s Program on Sudden Death in Pediatrics Boston Children’s Hospital Boston MA USA; ^3^ Department of Pediatrics University of Leuven Leuven Belgium; ^4^ Department of Pediatrics Boston Children’s Hospital and Harvard Medical School Boston MA USA; ^5^ Department of Medicine Division of Genetics and Genomics and the Manton Center for Orphan Disease Research Boston Children's Hospital Boston MA USA; ^6^ Genomic Medicine Institute Lerner Research Institute Cleveland Clinic Cleveland OH USA; ^7^ Cologne Center for Genomics University of Cologne Cologne Germany; ^8^ Department of Pathology Boston Children’s Hospital and Harvard Medical School Boston MA USA; ^9^ Stanley Center for Psychiatric Research Broad Institute of Harvard and MIT Cambridge MA USA

**Keywords:** arrhythmia, epilepsy, sodium channel, sudden unexpected death

## Abstract

**Background:**

Sudden Unexpected Death in Pediatrics (SUDP) is a tragic event, likely caused by the complex interaction of multiple factors. The presence of hippocampal abnormalities in many children with SUDP suggests that epilepsy‐related mechanisms may contribute to death, similar to Sudden Unexplained Death in Epilepsy. Because of known associations between the genes *SCN1A* and *SCN5A* and sudden death, and shared mechanisms and patterns of expression in genes encoding many voltage‐gated sodium channels (VGSCs), we hypothesized that individuals dying from SUDP have pathogenic variants across the entire family of cardiac arrhythmia‐ and epilepsy‐associated VGSC genes.

**Methods:**

To address this hypothesis, we evaluated whole‐exome sequencing data from infants and children with SUDP for variants in VGSC genes, reviewed the literature for all SUDP‐associated variants in VGSCs, applied a novel paralog analysis to all variants, and evaluated all variants according to American College of Medical Genetics and Genomics (ACMG) guidelines.

**Results:**

In our cohort of 73 cases of SUDP, we assessed 11 variants as pathogenic in *SCN1A, SCN1B,* and *SCN10A*, genes with long‐standing disease associations, and in *SCN3A, SCN4A,* and *SCN9A*, VGSC gene paralogs with more recent disease associations. From the literature, we identified 82 VGSC variants in SUDP cases. Pathogenic variants clustered at conserved amino acid sites intolerant to variation *across* the VGSC genes, which is unlikely to occur in the general population (*p* < .0001). For 54% of variants previously reported in literature, we identified conflicting evidence regarding pathogenicity when applying ACMG criteria and modern population data.

**Conclusion:**

We report variants in several VGSC genes in cases with SUDP, involving both arrhythmia‐ and epilepsy‐associated genes. Accurate variant assessment as well as future studies are essential for an improved understanding of the contribution of sodium channel‐related variants to SUDP.

## Graphical abstract

Sudden Unexpected Death in Pediatrics (SUDP) is a tragic event. We evaluated WES data from 73 cases and reviewed the literature for sodium channel variants in SUDP. We report variants in several sodium channel genes, involved in central nervous system and/or cardiac rhythm dysfunction.

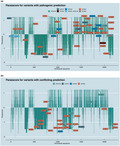

## 1 INTRODUCTION

Sudden Unexpected Death in Pediatrics (SUDP) encompasses a tragic set of conditions, including Sudden Infant Death Syndrome (SIDS) and Sudden Unexplained Death in Childhood (SUDC), affecting children under and over 1 year of age, respectively (Goldstein, Nields, & Kinney, 2017). These conditions are hypothesized to involve heterogeneous and multifactorial etiologies, conceptualized as a ‘triple‐risk’ model with a convergence of intrinsic, developmental, and environmental vulnerabilities contributing to death (Filiano & Kinney, 1994; Goldstein, Kinney, & Willinger, 2016).

Pathogenic variants in both arrhythmia‐ and epilepsy‐related voltage‐gated sodium channels (VGSCs) have been identified in association with sudden death, including SIDS, SUDC, and the related phenomenon Sudden Unexpected Death in Epilepsy (SUDEP) (Arnestad et al., 2007; Baruteau, Tester, Kapplinger, Ackerman, & Behr, 2017; Brownstein et al., 2018; Glengarry et al., 2014; Halvorsen et al., 2016; Howell et al., 2015; Johannesen et al., 2018; Kapplinger et al., 2010; Kato et al., 2014; Millat et al., 2009; Otagiri et al., 2008; Plant et al., 2006; Priori, Napolitano, Giordano, Collisani, & Memmi, 2000; Tester & Ackerman, 2005; Turillazzi et al., 2008; Veeramah et al., 2012; Wang et al., 2014; Wedekind et al., 2001; Winkel et al., 2015). We have reported a range of developmental hippocampal malformations in greater than 40% of children with SIDS and SUDC (Kinney et al., 2015, 2016), including hippocampal lesions such as dentate bilamination that have been classically associated with temporal lobe epilepsy (Houser, 1990). These observations suggest that a subset of SIDS and SUDC is linked to epilepsy‐related mechanisms. The association between epilepsy and sudden death, demonstrated most clearly in Sudden Unexpected Death in Epilepsy (SUDEP), may well extend to SUDP in cases with these hippocampal lesions in the absence of a history of overt epilepsy, conceptualized as ‘epilepsy in situ’ (Noebels, 2016) since infants and children with SIDS and SUDP by definition have not presented with a history of epilepsy. An example of a gene with many protean disease associations that has been associated with SIDS, SUDC, and SUDEP is *SCN1A* [MIM *182389], providing a specific contributing factor that links these entities (Bagnall et al., 2016; Bagnall, Crompton, & Semsarian, 2017; Brownstein et al., 2018; Halvorsen et al., 2016).

While the terminal mechanisms of SUDP, including SUDEP, remain speculative (Devinsky, Hesdorffer, Thurman, Lhatoo, & Richerson, 2016; Massey, Sowers, Dlouhy, & Richerson, 2014), there is active investigation into the role of genetic factors involving genes related to epilepsy (Brownstein et al., 2018; Goldman et al., 2009) as well as cardiac arrhythmia (Bagnall et al., 2016; Baruteau et al., 2017) that may contribute to these untimely deaths. Given the identification of pathogenic *SCN1A* variants in individuals with SIDS, SUDC, and SUDEP, and given the known robust association between *SCN5A* [MIM *600163] and sudden death (Brugada syndrome), we sought to identify additional variants across the entire VGSC family of genes that encode for several brain‐ and cardiac‐expressed genes. The VGSCs are a highly conserved family of proteins – expressed in excitable tissue in the heart, central nervous system, peripheral nervous system, and muscle – that are essential for the generation and propagation of action potentials. In humans, nine different pore‐forming α‐subunits have been identified (Na_V_1.1‐1.9 encoding for *SCN1A‐SCN5A* [MIM: *SCN2A* *182390; *SCN3A* *182391; *SCN4A* *603967] and *SCN8A‐SCN11A* [MIM: *SCN8A* *600702; *SCN9A* *603415; *SCN10A* *604427]) (Catterall, 2014). Na_V_1.1, 1.2, 1.3, and 1.6 are the primary sodium channel subunits expressed in the central nervous system, Na_V_1.7, 1.8, and 1.9 in the peripheral nervous system, Na_V_1.4 in skeletal muscle, and Na_V_1.5 in the heart. The pore‐forming α‐subunit is composed of four homologous domains, each containing six transmembrane α‐helical segments (S1–S6). In addition, there are five β‐subunits (β1, β1B, β2, β3, and β4) encoded by *SCN1B‐SCN4B* [MIM: *SCN1B* *600235; *SCN2B* *601327; *SCN3B* *608214; *SCN4B* *608256] (Brackenbury & Isom, 2011). The tissue‐specific expression profiles of α‐subunits and β‐subunits are shown in Table 1. Variants in the cardiac‐expressed gene *SCN5A* (Arnestad et al., 2007; Baruteau et al., 2017; Glengarry et al., 2014; Kato et al., 2014; Millat et al., 2009; Otagiri et al., 2008; Plant et al., 2006; Priori et al., 2000; Tester & Ackerman, 2005; Turillazzi et al., 2008; Wang et al., 2014; Wedekind et al., 2001; Winkel et al., 2015) have been reported in association with SIDS and SUDC. Variants in other VGSCs are only rarely identified in cases with SIDS and SUDC: *SCN1A* (Brownstein et al., 2018; Halvorsen et al., 2016), *SCN4A* (Männikkö et al., 2018), *SCN10A* (Neubauer et al., 2017)*, SCN1B* (Altshuler et al., 2012; Baruteau et al., 2017; Denti, n.d.; Hu et al., 2012; Neubauer et al., 2017), *SCN3B* (Tan et al., 2010; Winkel et al., 2015)*,* and *SCN4B* (Tan et al., 2010). Variants in epilepsy‐associated VGSC genes expressed in the central nervous system have also been associated with SUDEP: *SCN1A* (Cooper et al., 2016; Gal et al., 2010), *SCN2A* (Howell et al., 2015; Myers et al., 2018)*,* and *SCN8A* (Johannesen et al., 2018; Myers et al., 2018; Veeramah et al., 2012).

**TABLE 1:** Voltage‐gated sodium channels expression and disease associations

Gene.	Protein	Distribution	Associated human diseases
*SCN1A*	Nav1.1	CNS, heart	DS, GEFS+, FS+, familial autism, FHM3, SUDEP
*SCN2A*	Nav1.2	CNS	DS, GEFS+, OS, EOEE, BFNIS
*SCN3A*	Nav1.3	CNS, heart	Unclear, contributing to neuronal hyperexcitability/ epilepsy?
*SCN4A*	Nav1.4	Skeletal muscle	PAM, PMC, HyperPP, HypoPP, SNEL
*SCN5A*	Nav1.5	Skeletal muscle, heart, CNS	AF, AS, BS, DCM, LQTS, PCCD, SIDS, SSS, SUDEP
*SCN8A*	Nav1.6	CNS, PNS	EOEE, cognitive impairment, paralysis, ataxia, dystonia
*SCN9A*	Nav1.7	PNS	CIP, IEM, PEPD, PPN
*SCN10A*	Nav1.8	PNS	PPN
*SCN11A*	Nav1.9	PNS	PPN
*SCN1B*	β1	CNS, PNS, heart, skeletal muscle	AF, BS*, DS, GEFS+, PCCD, TLE
*SCN2B*	β2	CNS, PNS, heart, skeletal muscle	AF, BS*
*SCN3B*	β3	CNS, PNS, heart, skeletal muscle	AF, BS*, PCCD, SIDS, ventricular fibrillation
*SCN4B*	β4	CNS, PNS, heart, skeletal muscle	LQTS*, SIDS
*SCN1B*	β1B	Fetal CNS, PNS, heart, skeletal muscle	BS*, PCCD, epilepsy

Note

Modified with permission from Brunklaus, Ellis, Reavey, Semsarian, and Zuberi (2014).

Abbreviations: AF, Atrial fibrillation; AS, atrial standstill; BFNIS, benign familial neonatal‐infantile seizures; BS*, disputed evidence for BS (Hosseini et al., 2018);BS, Brugada syndrome; CIP, channelopathy‐associated insensitivity to pain; CNS, central nervous system; DCM, dilated cardiomyopathy; DS, Dravet syndrome; EOEE, early‐onset epileptic encephalopathy; FHM3, familial hemiplegic migraine type 3; FS+, febrile seizures plus; GEFS+, genetic epilepsy with febrile seizures plus; HyperPP, hyperkalemic periodic paralysis, HypoPP, hypokalemic periodic paralysis; IEM, inherited erythromelalglia; LQTS*, disputed evidence for LQTS (Adler et al., 2020); LQTS, long QT syndrome; OS, Ohtahara syndrome; PAM, potassium‐aggravated myotonia; PCCD, progressive cardiac conduction disease; PEPD, paroxysmal extreme pain disorder formally known as familial rectal pain syndrome; PMC, paramyotonia congenital; PNS, peripheral nervous system; PPN, painful peripheral neuropathies; SIDS, sudden infant death syndrome; SNEL, severe neonatal episodic laryngospasm; SSS, sick sinus syndrome; SUDEP, sudden unexplained death in epilepsy; TLE, temporal lobe epilepsy.

Given that VGSC genes are highly conserved in linear protein sequence and share essential functional domains across cardiac and neurologic tissues, we hypothesized a role for the entire family of VGSC genes in SUDP. We evaluated for the presence of variants in all VGSC genes using WES data from a cohort of 73 cases with SUDP; we applied a structure‐based assessment of all novel and reported variants in human VGSC in SUDP cases versus controls.

## 2 METHODS

### 2.1 Ethics statement

This study was approved by the Institutional Review Board of Boston Children's Hospital (approval number P00011014), and informed written consent has been obtained from the parents of all cases included. Some cases were ascertained through the San Diego SIDS Registry, which included consent for research but did not allow recontact of families and did not obtain parental DNA.

### 2.2 Our SUDP cohort

DNA from 73 SUDP cases was obtained through the Massachusetts Office of the Chief Medical Examiner (OCME), Boston, MA, and the Office of the Medical Examiner, San Diego, CA, using consent procedures in accordance with Massachusetts and California Law. These cases included 42 singletons, for whom parental samples were not available and for whom families could not be contacted, 28 trios consisting of probands and both parents, and 3 probands with one parent's sample available. All cases were sudden, unexpected deaths that remained unexplained after a complete autopsy and death scene investigation (Goldstein et al., 2017). DNA extracted from whole blood or saliva underwent capture for exome sequencing using either the Agilent SureSelect XTHuman All Exon v4 or Illumina Rapid Capture Exome enrichment kit (Broad Institute). Sequencing of 100 bp paired end reads was obtained using Illumina HiSeq (Illumina). Coverage was >90% or >80% meeting 20x coverage with the two methods, respectively. Our data analysis and variant calling methods have been described previously (Olson et al., 2017). We utilized the BCH (Boston Children`s Hospital) Connect Genomics Gateway integrated with the *WuXi NextCODE* analysis platform (Gudbjartsson et al., 2016) for variant interrogation and analysis.

For each case, we performed a targeted initial analysis to identify variants in genes encoding for the human VGSC subunits. Candidate pathogenic variants were evaluated according to American College of Medical Genetics and Genomics (ACMG) criteria (Richards et al., 2015), including pathogenicity predictions from both Polyphen2 and SIFT and low population allele frequency (<0.001) according to the Genome Aggregation Database (gnomAD, http://gnomad.broadinstitute.org) representing control individuals; while not all individuals in this database are disease‐free, they did not experience sudden death in infancy or childhood. Furthermore, given that the genes in question are known to have decreased penetrance, we did not require complete absence in gnomAD for pathogenicity. For cases with data from parental samples, we evaluated de novo versus inherited status of candidate variants of interest. For splicing variants, we used the splicing prediction score from *Alamut Visual‐2.10*, which incorporates the splicing tools MaxEnt, NNSPLICE, and HSF. We evaluated trios for de novo or inherited heterozygous variants, homozygous or compound heterozygous variants in any disease‐associated genes. We additionally evaluated all cases, including singletons, for potentially pathogenic variants in disease‐associated genes related to sudden death or cardiac arrhythmia.

### 2.3 Literature cohort

In order to identify additional cases for phenotypic comparison and to evaluate whether a given variant was novel or previously reported and whether there might be data supporting pathogenicity, we performed a literature search (PubMed, accessed June 2019, with search parameters “Sudden Infant Death” [Mesh] AND “Sodium Channels” [Mesh]) resulting in the identification of 49 studies. In addition, we searched the Human Gene Mutation Database (http://www.hgmd.cf.ac.uk/, accessed June 2019) for each of the VGSCs genes to identify any possible variants not found in the literature search, identifying 13 additional studies with cases of SIDS or SUDC and reported variants in sodium channel‐related genes.

### 2.4 Structural protein modeling

We used the human Na_V_1.7 (*SCN9A*) protein model described by Shen, Liu, Wu, Lei, and Yan (2019) and analyzed the position of the variants of our cohort along with additional variants identified through our literature search (Table S1). Three of our cases’ variants have been previously reported in literature (Brownstein et al., 2018; Halvorsen et al., 2016). We focused on exonic variants since intronic, splicing, and truncating variants cannot be annotated onto the three‐dimensional protein sequence. Illustrations were generated using PyMol.

### 2.5 In silico predictions

Functional prediction scores were obtained from the dbNSFP database version 3.5 (August 2017, http://varianttools.sourceforge.net/). In total, we used six pathogenicity prediction scores (SIFT, Polyphen‐2‐HVAR, Polyphen‐2‐HDIV, Mutation Assessor, FATHMM, and LRT). We classified a variant as “damaging” when the majority of the tools predicted a functional effect for the variant (i.e., a minimum of 4 of 6 tools). Splicing variants are considered “possibly damaging” or “damaging” when they have a likelihood of 50% or more to affect splicing, using the splicing prediction score from Alamut Visual‐2.10, calculated from the splicing tools MaxEnt, NNSPLICE, and HSF.

### 2.6 Parazscore

Based on the linear amino acid sequence of *SCN9A* (canonical transcript ENST00000409672, CCDS46441), we compared the position of 74 missense variants in all sodium channel alpha‐gene paralogs against variants found in the general population using gnomAD. We evaluated the amino acid gene‐family paralog conservation score using the Parazscore (Lal et al., 2017) (http://per.broadinstitute.org/), which leverages amino acid conservation across gene‐family members, assuming that conserved sites are more likely to be important for protein function and thus more likely to be present in cases than in controls (Brunklaus et al., 2020). Statistical comparison between the variant counts of cases versus gnomAD was conducted using a two‐tailed *t* test with nominal two‐sided *p*‐values < .05 considered significant.

### 2.7 Three‐dimensional mapping of amino acid substitutions in VGSC‐related genes

In order to assess for correlation across gene‐family paralogs, we compared the position of all missense variants in sodium channel alpha‐gene paralogs from our own cohort and from the literature onto a three‐dimensional Na_V_1.7 structure model. We used the human Na_V_1.7 structure model that is based on the cryo‐electron microscopy structures of the human Na_V_1.7‐ß1‐ß2 complex at overall resolutions of 3.2 angstroms (Shen et al., 2019).

## 3 RESULTS

### 3.1 Genetics and clinical characteristics of our cases

**FIGURE 1:**
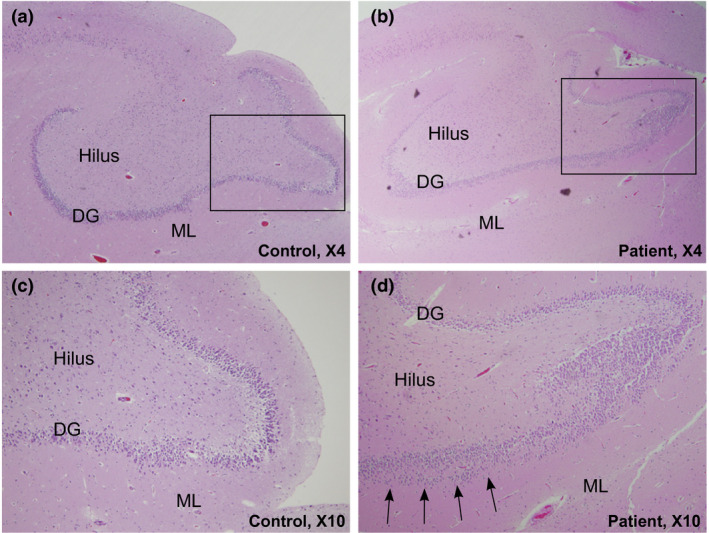
Hippocampal developmental lesions in an infant with SIDS (Case 6 with *SCN4A* c.307T>G, p.Phe103Val variant). (a) Control dentate gyrus in an age‐matched infant showing the normal single layer of dentate gyrus granule cells in a row rectangle (H&E, X4). (b) Low‐power photograph of the hippocampus shows an abnormal dentate gyrus with a region of focal bilamination highlighted in the black rectangle (H&E, X4). (c) Enlarged detail of control dentate gyrus (H&E, X10). (d) Enlarged detail of focal dentate gyrus bilamination with two layers of granule cells (H&E, X10. DG, dentate gyrus; H&E, hematoxylin & eosin stain; ML, molecular layer). SIDS, Sudden Infant Death Syndrome

In our cohort of 73 SUDP cases, we identified from exome data a total of 45 variants in VGSC genes, 11 of which (present in 10 cases) were predicted to be pathogenic or likely pathogenic (*n* = 6), or VUS (*n* = 5) applying ACMG criteria (Richards et al., 2015) (Table 2). No additional heterozygous, homozygous, or compound heterozygous variants were present that provided an explanation for sudden death in these individuals. Age of death across the 10 cases with pathogenic or likely pathogenic variants in VGSC genes ranged from 7 weeks to 8 years, with 9/10 (90%) individuals younger than 6 months at the time of death. Seven had hippocampal malformations as assessed by detailed neuropathological examination, and one had a normal hippocampus. For the other two, material was not available for detailed neuropathological analysis. Clinical and molecular data for all 10 cases are listed in Table 2. Neuropathological examination of an abnormal dentate gyrus is illustrated in Figure 1. Case 1 had two variants in *SCN1A* (p.Leu1296Met and p.Glu1308Asp) (reported previously) (Brownstein et al., 2018), Case 4 had a variant in *SCN3A* (p.Ala1804Val) and in *SCN10A* (c.4386+1G>C), and two siblings (Cases 9 and 10) carried the same variant in *SCN1B* (p.Trp179Ter) (unknown whether inherited from a parent with a germline or mosaic variant as parental DNA is unavailable). None of the cases in our cohort had a diagnosis of epilepsy. Case 3 had a history of atypical febrile seizures. Cases 5 and 6 each had an inherited variant in *SCN4A* (p.Lys724Arg and p.Phe103Val, respectively); neither variant‐bearing parent had reported history of muscle disease or muscle twitching. None of the individuals in our cohort was reported to have had cardiac arrhythmia. We did not identify pathogenic variants in the cardiac‐expressed *SCN5A* gene in our cohort or in the epilepsy‐associated genes *SCN2A* and *SCN8A*.

**TABLE 2:** Variants in voltage‐gated sodium channel genes in our cohort

Gene	Case	cDNA, protein	AF	Pathogenicity prediction	Parazscore	ACMG	Inheritance, zygosity	Age	HC	Additional notes
*SCN1A*	1	3886T>A, L1296M	8.17E‐06	PP‐2, 0.979; SIFT, 0.001	0.49	P	N/A, het, in *cis* with E1308D	7 weeks	Abnormal DG (Brownstein et al., 2018)	
	1	3924A>T, E1308D	0.0006416	PP‐2, 0.727; SIFT, 0.281	−1.23	LP	N/A, het, in *cis* with L1296M	7 weeks		
	2	2045G>T, G682V	4.07E‐06	PP‐2, 0.478; SIFT, 0.003	−1.48	LP	N/A, het	2 months	Abnormal DG (Brownstein et al., 2018)	
	3	182T>C, L61P	0	PP‐2, 0.783; SIFT, 0	1.23	LP	N/A, het	20 months	End‐folium sclerosis	Atypical FS
*SCN3A*	4	5411C>T, A1804V	2.44E‐05	PP‐2, 0.998; SIFT, 0	−0.49	VUS	N/A, het	4 months	N/A	
*SCN4A*	5	2171A>G, K724R	0	PP‐2, 0.953; SIFT, 0.001	0.25	P	Inh, het	5 months	Normal	Megalencephaly, bilateral open opercula, bilateral small STG, chronic hemorrhages, acute HIE
	6	307T>G, **F103V**	2.03E‐05	PP‐2, 0.73; SIFT, 0.001	−0.25	VUS	Inh, het	3 months	Abnormal DG	Megalencephaly, mild gliosis of CWM, CerWM, inferior olive, tegmentum
*SCN9A*	7	5624G>A, R1875Q	1.62E‐05	PP‐2, 0.734; SIFT, 0	0	VUS	Inh, het	8 years	Abnormal DG	Cytomegaly in RF, Chiari malformation 1
*SCN10A*	4	4,386 + 1G>C	1.22E‐05	splicing (100%)	N/A	VUS	N/A, het	4 months	N/A	
	8	305C>G, **S102C**	4.07E‐06	PP‐2, 0.701; SIFT, 0.002	−0.25	VUS	Inh, het	3 months 22 days	Abnormal DG	Megalencephaly
*SCN1B*	9	536G>A, W179*	8.19E‐06	nonsense	N/A	LP	N/A, het	4 months	Abnormal DG	Megalencephaly
	10	536G>A, W179*	8.19E‐06	nonsense	N/A	LP	N/A, het	2 months	N/A	Megalencephaly

Note

For splicing variants, we used the splicing prediction score from Alamut Visual‐2.10, which incorporates the splicing tools MaxEnt, NNSPLICE, and HSF. Variants highlighted in **bold** affect the same paralog position. Cases 1, 2, and 3 have been reported previously (Brownstein et al., 2018; Halvorsen et al., 2016). Cases 9 and 10 are siblings.

Transcripts used: *SCN1A* (NM_001165963.1), *SCN3A* (NM_0006922.3), *SCN4A* (NM_000334.3), *SCN5A* (NM_198056.2), *SCN9A* (NM_002977.3), *SCN10A* (NM_006514.2), *SCN1B* (NM_199037.4), *SCN3B* (NM_018400.3), and *SCN4B* (NM_174934.3).

Abbreviations: ACMG, American College of Medical Genetics and Genomics; AF, allele frequency according to gnomAD; CerWM, cerebellar white matter; CWM, cerebral white matter; DG, dentate gyrus; FS, febrile seizures; HC, hippocampus; het, heterozygous; HIE, hypoxic ischemic encephalopathy; inh, inherited; LP, likely pathogenic; N/A, not available; P, pathogenic; PP‐2, Polyphen 2; RF, reticular formation; SIFT, Sorting Intolerant From Tolerant; STG, superior temporal gyrus; VUS, variant of unknown significance.

### 3.2 Cases from the literature

We reviewed all variants in VGSC genes that have been reported in the literature in infants and children with SIDS and SUDC. We identified 82 variants in 103 individuals, with 6 harboring two variants and 12 variants recurring in 39 cases, affecting 81 different amino acid positions in the following genes: *SCN1A* (*n* = 3), *SCN4A* (*n* = 6), *SCN5A* (*n* = 62), *SCN10A* (*n* = 4), *SCN1B* (*n* = 3), *SCN3B* (*n* = 3), and *SCN4B* (*n* = 1). The two variants recurring most frequently were identified in *SCN5A*: c.3578G>A (R1193Q) and c.1700T>A (L567Q), occurring in 8 and 7 cases, respectively.

### 3.3 Paralog conservation of pathogenic or likely pathogenic VGSC variants from our cohort and all variants in the literature

Collectively, we identified a total of 90 unique VGSC variants (Table S1), 11 from our cohort and 82 from the literature (3 overlapping) affecting 89 different amino acid positions in the following genes: *SCN1A* (*n* = 4), *SCN3A* (*n* = 1), *SCN4A* (*n* = 8), *SCN5A* (*n* = 62), *SCN9A* (*n* = 1), *SCN10A* (*n* = 6), *SCN1B* (*n* = 4), *SCN3B* (*n* = 3), and *SCN4B* (*n* = 1). Interestingly, variants in the epilepsy‐associated genes *SCN2A* and *SCN8A* were not observed in our cohort or in the SUDP literature cases.

**FIGURE 2:**
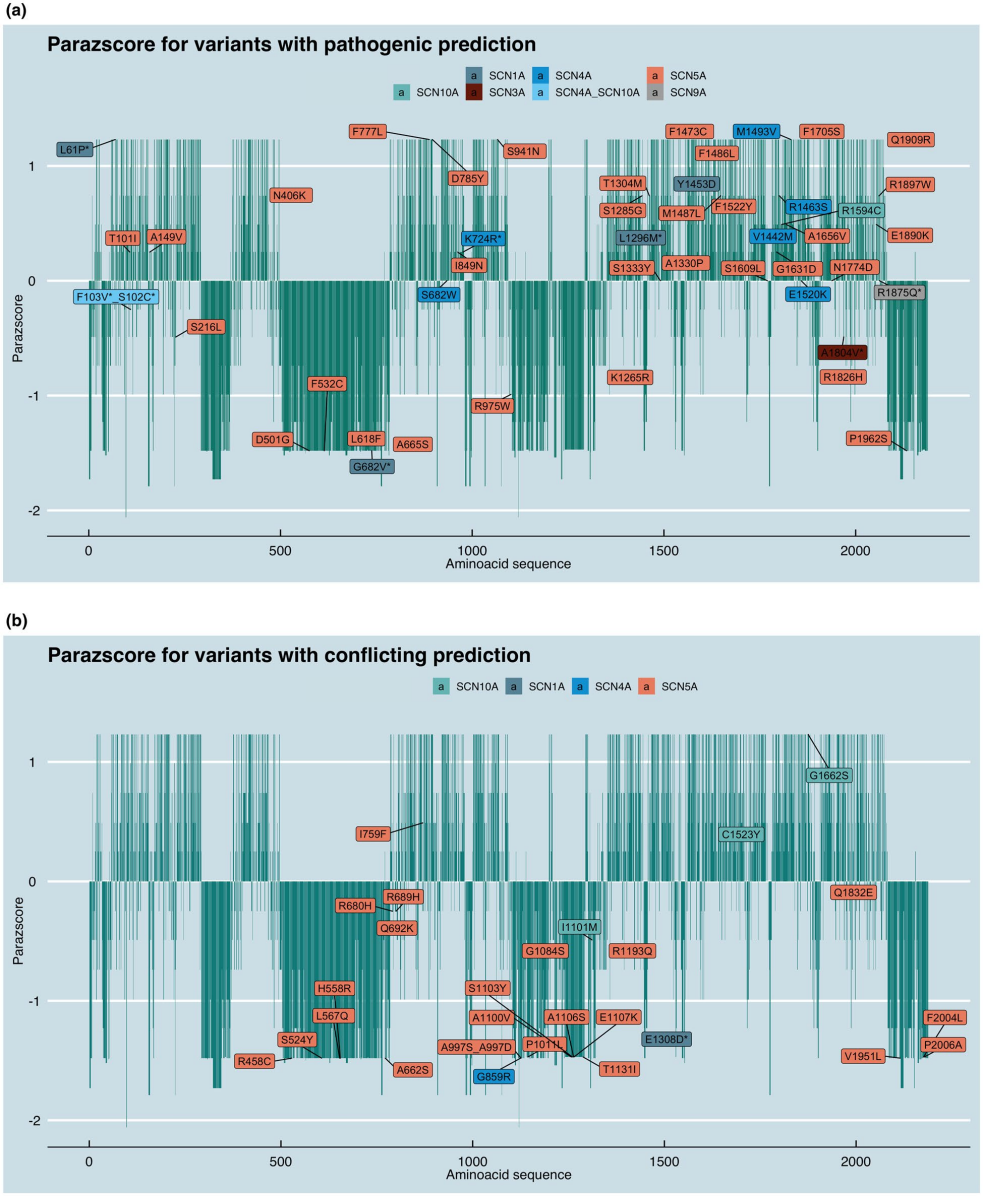
*SCN* case variant evolutionary conservation and population constrained assessment. The *SCN* case variant paralog conservation score (Parazscore) is shown across the linear protein sequence. Parazscore values range from negative values, representing less conservation at a given amino acid position, to positive values, representing high conservation, with the highest value depicting identical amino acids are present in all related proteins. Variants in our cohort are marked with a *. (a) The Parazscore is shown for *SCN* variants that are predicted to be pathogenic. (b) The Parazscore is shown for *SCN* variants that are predicted to be conflicting

To assess whether some regions of the VGSCs tolerate variation, we evaluated for clustering of variants in analogous locations across the VGSCs using the Parazscore (Lal et al., 2017). As expected, Parazscores from patient (case) variants were significantly higher than those observed in gnomAD (unpaired *t* test, *p* < .0001). Variants that were determined to be (likely) pathogenic with the prediction tools were more likely to be located at paralog conserved amino acid positions (Fisher exact, *p* = .03) (i.e., greater Parazscores) (Figure 2a). On the other hand, variants that we determined to be conflicting or benign with the prediction scores were more likely present in less conserved regions (Fisher's exact test, *p* = .0001) (Figure 2b). Interestingly, 25 variants involved the 'alignment index position' of a VGSC variant reported in disease (Table S2), which is unlikely to occur in the general population (*p* < .0001). Overall, we observed significant clustering of variants at conserved amino acid sites, notably with the same amino acid affected between *SCN1A/SCN5A* and *SCN5A/SCN9A* (Table S2). The variants of two cases (Cases 6 and 8) in our cohort affected the same paralog position: *SCN4A* (p.Phe103Val) and *SCN10A* (p.Ser102Cys) (*p* < .0001). Both had died at 3 months of age and had pathology notable for hippocampal granule cell dispersion with dentate gyrus bilamination (Table 2); both variants were inherited from a parent who had no history of epilepsy, febrile seizures, or other major illness. Two other cases from our cohort (Cases 1 and 7) had variants affecting the same paralog position at *SCN1A/SCN5A* and *SCN5A/SCN9A*, respectively. Interestingly, neuropathological examination in both cases revealed dentate gyrus bilamination (Figure 1, Table 2 and Table S2).

### 3.4 Re‐evaluation of cases from the literature

Given our observation that several variants, particularly those from the literature, had conflicting or benign scores, we re‐assessed the 82 variants from the literature using the same criteria applied to our cases; we classified 30 variants in 35 cases, with 5 variants occurring in multiple individuals, as pathogenic or likely pathogenic using ACMG criteria (Richards et al., 2015) (Table S1). Evaluation of variants in the literature with respect to allele frequency reported in the gnomAD database revealed 14/82 variants (17%) in 31/103 individuals (30%) with an allele frequency higher than or equal to 0.001, arguing against their pathogenicity. Thirteen variants in 30 cases were classified as benign or likely benign using ACMG criteria.

In addition, we also evaluated published functional studies and used six pathogenicity prediction scores to classify the variants. Of the 82 variants reported in literature, we determined that 34 variants (34/82 = 41%) in 55 individuals (55/103 = 53%) had conflicting evidence of pathogenicity, and 1 of 82 (1%) was determined to be benign based on the high frequency in controls, lack of predicted functional effect in silico*,* and/or in vivo absence of functional effects resulting from the variants (Table S1). Notably, the two most commonly recurring variants in *SCN5A* (c.3578G>A, R1193Q and c.1700T>A, L567Q) were assessed as benign using ACMG criteria and as conflicting according to pathogenicity prediction scores and functional studies. Overall, for 56/103 (54%) variants reported in literature in cases of sudden death, we identified conflicting evidence of pathogenicity.

### 3.5 Variant position and pathogenicity

Variants predicted to be pathogenic were more likely to be localized in the transmembrane regions of the protein than variants predicted to be conflicting or benign (*p* = .03). Variants localized in the transmembrane regions have been associated with severe channel dysfunction (Wallace et al., 2003) (Table S1, Figure S1).

## 4 DISCUSSION

From our cohort of 73 cases with SUDP, we identified 11 variants predicted to be pathogenic or likely pathogenic (*n* = 6), or VUS (*n* = 5) in genes encoding for VGSC subunits expressed in the brain and/or in the heart, including two individuals who each had 2 variants. Among the 11 cases, 7 had hippocampal abnormalities but no history of epilepsy, 1 (Case 3) had a history of atypical febrile seizures. None had a history of cardiac arrhythmia or other cardiac presentation prior to death. While *SCN1A* (Brownstein et al., 2018; Halvorsen et al., 2016) and *SCN1B* (Altshuler et al., 2012; Baruteau et al., 2017; Denti, n.d.; Neubauer et al., 2017) have strong prior associations with epilepsy, arrhythmia, and sudden death, and *SCN4A* (Männikkö et al., 2018) a recent association with sudden death, we additionally observed variants in the *SCN* paralogs *SCN3A, SCN9A,* and *SCN10A* that have yet to be conclusively associated with sudden death. Surprisingly, we did not identify pathogenic or likely pathogenic variants in *SCN5A* in our cohort; this contrasts with the 84/103 (82%) of SIDS cases reported in literature with variants in this gene, but we also observed that 47/84 (56%) of *SCN5A* variants reported in literature had conflicting evidence for pathogenicity when evaluated using modern data and criteria. On the other hand, 4/12 (33%) of our cohort's variants compared to 3/82 (4%) variants identified in cases from the literature carried a variant in *SCN1A*, three of them recently reported (Brownstein et al., 2018; Halvorsen et al., 2016). It is possible that these differences are due to differences in ascertainment as well as differences between past and current methods of variant assessment. Notably, we did not identify variants within *SCN2A* and *SCN8A* genes, two genes previously associated with epilepsy and SUDEP (Howell et al., 2015; Myers et al., 2018).

We identified in our literature review 82 variants in 103 cases with SIDS, SIDS‐like presentations, or SUDC affecting 81 different amino acid positions in a VGSC‐related gene. The majority of variants (62/82, 76%) were present in *SCN5A*, reflecting in some cases the fact that this gene was specifically targeted in some series (Ackerman et al., 2004; Glengarry et al., 2014; Kato et al., 2014; Millat et al., 2009; Otagiri et al., 2008; Wang et al., 2014; Wedekind et al., 2001; Winkel et al., 2015). Since many of the VGSC variants reported in SIDS or SUDC were described before the current era of variant interpretation guidelines, we re‐evaluated all the variants reported in literature with this in mind. Next‐generation sequencing resulted in an exponential growth of identified variants, but clinical classification of variants is still in its early stages. In a surprisingly high number of reported variants (54%), conflicting evidence argued against pathogenicity using current ACMG criteria. While these results may challenge the conclusions of individual studies, correct classification and reclassification of variants based on evolving supportive evidence is important over time.

Analysis of the relatively modest number of variants in our cohort, coupled with a larger number from the literature, suggested that those variants we determined to be pathogenic or likely pathogenic by other metrics were most likely to be located in critical domains of the sodium channel protein – for example, transmembrane domains. Alternatively, variants in control populations were randomly distributed throughout the genes.

Variants considered pathogenic are enriched in conserved regions across gene family members. We had hypothesized that variants in VGSC genes at certain conserved positions might be associated with a risk for sudden death, independent of the tissue where the relevant gene is most highly expressed. This is illustrated by two cases in our cohort, each with a variant in *SCN4A* or *SCN10A* at the same paralog position (Cases 6 and 8) and 24 other variants from the literature that affect a paralog variant that is known to be disease associated (Table S2). When considering variant pathogenicity in a newly implicated VGSC gene, the position in the protein, with respect to paralogous proteins already implicated in sudden death, can provide additional evidence suggesting pathogenicity.

Ultimately, while all of these factors are taken into consideration when assessing pathogenicity, robustly conducted experimental evidence with adequate positive and negative control data should ultimately be sought when there is question regarding pathogenicity. While *SCN5A* (Arnestad et al., 2007; Baruteau et al., 2017; Glengarry et al., 2014; Kato et al., 2014; Millat et al., 2009; Otagiri et al., 2008; Plant et al., 2006; Priori et al., 2000; Tester & Ackerman, 2005; Turillazzi et al., 2008; Wang et al., 2014; Wedekind et al., 2001; Winkel et al., 2015), *SCN1B* (Altshuler et al., 2012; Baruteau et al., 2017; Denti, n.d.; Hu et al., 2012; Neubauer et al., 2017), and more recently *SCN1A* (Brownstein et al., 2018; Halvorsen et al., 2016) have been considered SUDP‐associated genes, larger cohort studies will be required to more securely implicate the broader range of VGSC‐related genes as part of the multirisk model leading to SUDP. In addition, future studies that can incorporate trio sequencing, in which parental DNA can be made available to determine whether variants are de novo and thus more likely to be pathogenic with respect to a severe phenotype like sudden death, will contribute to our understanding of the role of this family of genes to sudden death. Initial studies in induced pluripotent stem cell (iPSC)‐derived neurons and mouse models of *SCN1A*, traditionally associated with epilepsy, suggested cardiac and/or respiratory mechanisms of death (Auerbach et al., 2013; Kim et al., 2018). Additional in vitro and in vivo studies of the sodium channel gene family will move us toward understanding the mechanisms through which variants in sodium channel‐encoding genes contribute to sudden death.

## 5 CONCLUSIONS

Our analysis of a SUDP cohort and the present literature on sodium channel variants in SUDP cases, using population‐ and protein structure‐based predictive models, revealed variants across the VGSC subunit family of sodium channel genes. Importantly, in our cohort, these variants were identified in children without prior histories of epilepsy or unprovoked seizures, and only one with a history of seizures that were atypical febrile seizures, yet with hippocampal abnormalities and without history or family history of cardiac arrhythmia. In addition, using recent guidelines for variant interpretation, we observed that the majority of SUDP variants reported in the previous literature do not have enough evidence to be classified as pathogenic. Variant assessment using current ACMG guidelines will improve variant interpretation and prediction of pathogenicity.

Overall, similar to prior studies, variants predicted to be pathogenic were more likely to cluster at conserved amino acid sites intolerant to variation, in the individual genes and across the VGSC genes. Therefore, the position of a variant in a VGSC gene, with respect to paralogous proteins already implicated in SUDP, can provide clues toward underlying pathogenicity. These findings provide evidence that sodium channel abnormalities contribute to the complex phenotype in SUDP involving central nervous system and/or cardiac rhythm dysfunction. Future functional studies into the function of the sodium channels will elucidate the mechanisms through which variants in these genes underlie some cases of sudden death and may contribute to a framework in which early testing can be used to stratify infants and children at risk.

## CONFLICT OF INTERESTS

The authors have declared that no competing interests exist.

## AUTHOR CONTRIBUTIONS

A.M.R., R.D.G., and A.H.P. contributed to the conception and design of the study; all authors were involved in the analysis and interpretation of the data, and/or drafting the article or revising it critically.

## Supporting information

Fig S1

Table S1‐S2

## Data Availability

Raw data were not deposited but may be available by contacting the authors.
