# Supplementary figures and images for: The role of sodium channels in sudden unexpected death in pediatrics
Source: Mol Genet Genomic Med. 2020 May 25;8(8):e1309. doi: 10.1002/mgg3.1309 (PMC7434613; doi:10.1002/mgg3.1309)

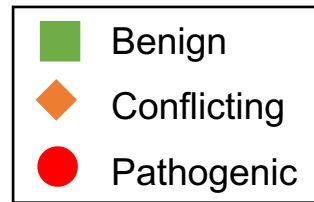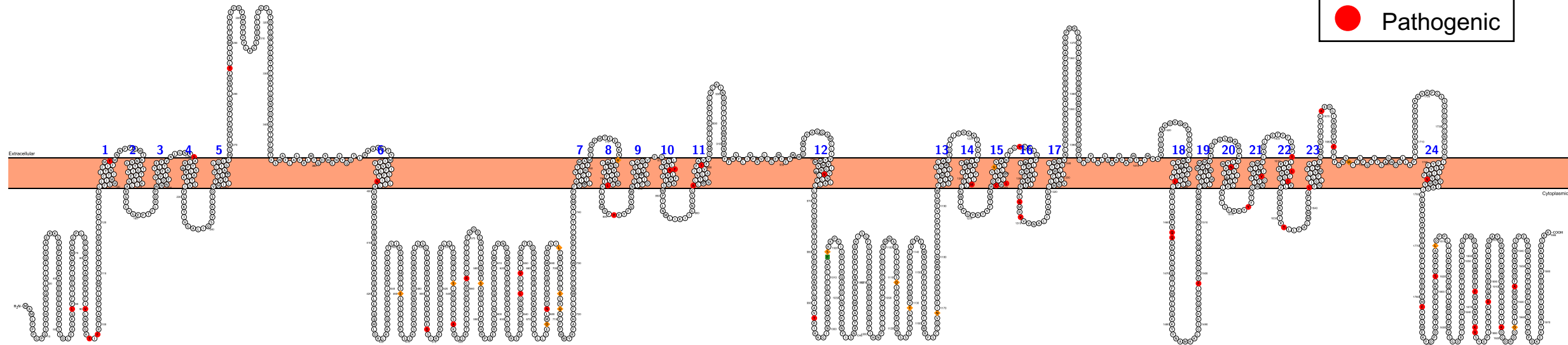

Supplement: Supplementary file 1 — Fig S1 [file MGG3-8-e1309-s001.pdf]
